# The *Scirtothrips dorsalis* Species Complex: Endemism and Invasion in a Global Pest

**DOI:** 10.1371/journal.pone.0123747

**Published:** 2015-04-20

**Authors:** Aaron M. Dickey, Vivek Kumar, Mark S. Hoddle, Joe E. Funderburk, J. Kent Morgan, Antonella Jara-Cavieres, Robert G. Jr. Shatters, Lance S. Osborne, Cindy L. McKenzie

**Affiliations:** 1 Mid-Florida Research & Education Center, University of Florida, Apopka, Florida, United States of America; 2 US Horticultural Research Laboratory, Fort Pierce, Florida, United States of America; 3 Department of Entomology, University of California, Riverside, California, United States of America; 4 Center for Invasive Species Research, University of California, Riverside, California, United States of America; 5 North Florida Research & Education Center, University of Florida, Quincy, Florida, United States of America; 6 U.S. Department of Agriculture, Agricultural Research Service, Fort Pierce, Florida, United States of America; 7 Indian River Research and Education Center, University of Florida, Fort Pierce, Florida, United States of America; Onderstepoort Veterinary Institute, SOUTH AFRICA

## Abstract

Invasive arthropods pose unique management challenges in various environments, the first of which is correct identification. This apparently mundane task is particularly difficult if multiple species are morphologically indistinguishable but accurate identification can be determined with DNA barcoding provided an adequate reference set is available. *Scirtothrips dorsalis* is a highly polyphagous plant pest with a rapidly expanding global distribution and this species, as currently recognized, may be comprised of cryptic species. Here we report the development of a comprehensive DNA barcode library for *S*. *dorsalis* and seven nuclear markers via next-generation sequencing for identification use within the complex. We also report the delimitation of nine cryptic species and two morphologically distinguishable species comprising the *S*. *dorsalis* species complex using histogram analysis of DNA barcodes, Bayesian phylogenetics, and the multi-species coalescent. One member of the complex, here designated the South Asia 1 cryptic species, is highly invasive, polyphagous, and likely the species implicated in tospovirus transmission. Two other species, South Asia 2, and East Asia 1 are also highly polyphagous and appear to be at an earlier stage of global invasion. The remaining members of the complex are regionally endemic, varying in their pest status and degree of polyphagy. In addition to patterns of invasion and endemism, our results provide a framework both for identifying members of the complex based on their DNA barcode, and for future species delimiting efforts.

## Introduction

Thrips (order Thysanoptera) are well represented in national inventories of invasive species suggesting that many species have a high propensity for invasion. Reasons for this include their small size (~1mm) and tendency to avoid detection by hiding in small crevices within plants [1]. Chilli thrips *Scirtothrips dorsalis* Hood (Thysanoptera: Thripidae), is a recent invader in the US with a rapidly expanding global distribution [2]. This highly polyphagous plant pest causes damage via direct feeding and indirect damage through disease transmission. Important crop hosts include tea, cotton, mango, citrus, rose, grapes, peanut, and pepper [3].

There is unanimity in the literature that *S*. *dorsalis* is native to portions of South Asia though for areas beyond extending to East Asia, Oceana, Australia, and South Africa many statements in the literature regarding its native range are contradictory or ambiguous. *S*. *dorsalis* was originally described as a pest of chilies and castor in India in 1919 [2]. Seal et al. in 2006 reported it as originally from South Asia [4] and Kumar et al. in 2013 reported it as native to the Indian subcontinent [3]. It was proposed by Mound and Palmer in 1981 to be native from Pakistan in the west to Japan, the Solomon Islands, and Queensland in the East however those authors believed that it was possible for it to have been confused with *Drepanothrips reuteri* in Japan [5]. *S*. *dorsalis* was reported for the first time in South Africa in 1986 [6] and Hoddle et al. reported it as native to Australasia and South Africa in 2008 [7]. In contrast, CABI in 2013 reported it to be native to Bangladesh, Myanmar, Pakistan, Sri Lanka, Taiwan, Thailand, Papua New Guinea, and the Solomon Islands but introduced in South Africa [2]. Multiple authors have also suggested that *S*. *dorsalis* may represent a cryptic species complex containing three-or-more morphologically indistinguishable species [7–8]. This suggestion further complicates an already disarrayed understanding of the native distribution of this pest. The range of *S*. *dorsalis* has undergone rapid expansion since the late 1990’s and now includes portions of the Middle East (Saudi Arabia, Israel); Central Africa (Côte d’Ivoire, Uganda); and the New World (USA, Barbados, Jamaica, Saint Lucia, Saint Vincent and the Grenadines, Trinidad, and Venezuela) [2].

Almost nothing is known about the genetic diversity within *S*. *dorsalis* (*sensu latu*) populations. Most genetic studies of the species have focused on developing molecular identification reactions via PCR and/or restriction fragments [8–12] with generally ≤2 individuals per population sequenced at a given locus. However, Kadirvel *et al*. [13] sequenced 19 individuals from two Indian populations at the mitochondrial cytochrome oxidase I (COI) locus and found 14 unique haplotypes. In another study, Toda *et al*. [10] sequenced the COI gene across an average of four individuals per population in 33 Japanese populations and found 34 unique native and invasive haplotypes. In the latter study, native genetic diversity was much higher than invasive diversity and invasive haplotypes were limited to a few crops and localities. Both these studies suggest the potential for using COI to describe genetic diversity in *S*. *dorsalis* populations. Because genetic diversity is often reduced in invasive populations relative to native populations due to founder effects and bottlenecks [14], discriminating between high and low diversity populations can be used to infer whether populations are native or invasive. The COI gene has also been used extensively to infer invasion pathways and to pinpoint the origin of an invading population [15–16]. Lastly, the 5’ end of the COI gene is widely used as a metazoan DNA barcode for identifying species [17–18], but this approach requires development of an extensive barcode library.

If *S*. *dorsalis* is a cryptic species complex, how many species does it contain and what should be the basis for delimiting species within the complex? Within Thysanoptera, the presence of cryptic species has been suggested for several species [7,8,11,13], but such species have been delimited only twice. Mound *et al*. [19] used a combination of mitochondrial COI sequence and morphology to describe the biological control agent *Pseudophilothrips gandolfoi* as distinct from *P*. *ichini*. In a second case, Rugman-Jones *et al*. [20] used congruence between the nuclear gene 28S D2 domain and the mitochondrial COI as the basis for separating two cryptic species of *Frankliniella occidentalis*. Recently, a variety of new methods have been developed for delimiting species with molecular barcode data [21] and with the addition of nuclear loci, the use of the multispecies coalescent may be useful for resolving contentious species boundaries [22].

The goals of this study were to construct a phylogenentic hypothesis of *Scirtothrips dorsalis* and to infer whether the origin of the invasive US population might be South Asia. However, due to the uncertainties regarding its native range and cryptic species status, it was determined that 1) a suitably comprehensive DNA barcode library, 2) genetic diversity estimates and phylogeographic analyses, and 3) appropriate species delimitation methods were needed to accomplish these goals. Herein we report significant progress in the development of a comprehensive DNA barcoding reference data set for *S*. *dorsalis*. This dataset was used to describe the genetic diversity within the *S*. *dorsalis* species complex and make inferences about patterns of invasion, endemism, host-use, virus transmission, and the global sources of invasive populations. Additionally, we report the development of nuclear genetic resources via next-generation sequencing. These nuclear loci were used in combination with the metazoan barcode to delimit species boundaries within the complex and to construct a robust molecular phylogeny. It was determined that a minimum of eleven species comprise the *S*. *dorsalis* complex and that three are invasive. The invasion risk these species pose to areas with compatible climates should be elevated based on their history of invasion [23].

## Methods

### Barcoding

**Fig 1:**
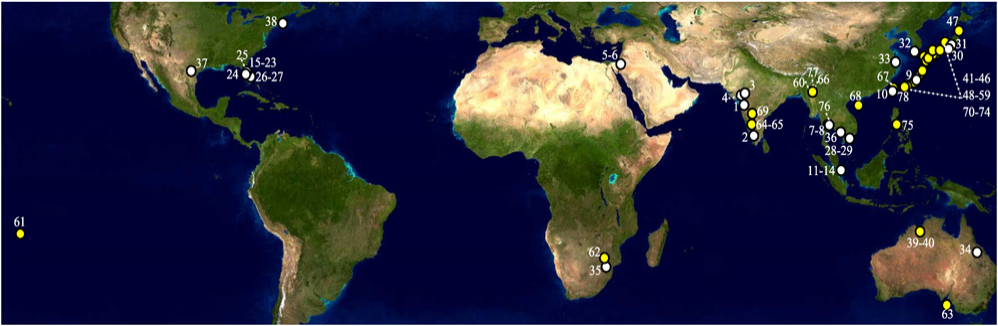
*Scirtothrips* locations sampled for this study (white, Table A of S1 File) or data mined (yellow, Table B of S1 File). The earth global view used in this figure consists of MODIS satellite data for the NASA Blue Marble 2002 project. These images are freely available to educators, scientists, museums, and the public.

Specimens were collected in the field and solicited from local, national, and international cooperators (Fig 1, Table A of S1 File). In addition, unique *S*. *dorsalis* barcodes not detected in our study were downloaded from GenBank (Fig 1, Table B of S1 File). All private landowners provided permission to collect. In instances of collection on public lands, no specific permissions were required as studies did not involve endangered or protected species. DNA was extracted from individuals using the DNEasy Blood & Tissue kit (Qiagen Inc., Valencia, CA), or by boiling in lysis buffer [24]. For at least one collection per country, DNA was extracted from a subset of individuals non-destructively by soaking insects overnight in lysis buffer and removing the carcass for permanent slide mount vouchering prior to completing the DNEasy extraction protocol. However, some larval and small adult carcasses did not yield vouchers of sufficient quality and population 8 from Thailand consisted only of DNA samples from a prior study [12]. Vouchers were prepared by JEF to be deposited in the Florida State Collection of Arthropods, Gainesville (Table A of S1 File). Each was examined for consistency with the following characters that are useful in separating *S*. *dorsalis* from other species in the genus: posterior fringe cilia on forewings straight, ocellar setae III between rear ocelli, abdominal tergites with brown antecostal ridge, and microtrichial field on abdominal tergites IV and V with 3 to 4 setae [5,25]. Initially the 655nt barcode region of COI was amplified using the primers LCO1490 and HCO2198 [26] for *S*. *dorsalis* from Florida, Singapore, Israel, and Japan. However PCR with these primers failed to produce a product for individuals from India, China, and Australia so additional primers were designed using the strategies of Simon *et al*. [27] and Ivanova *et al*. [28]. Thysanopterans have a highly rearranged mitochondrial genome relative to the ancestral arthropod [29] with the ribosomal RNA large subunit gene preceding COI so several primers were also designed that spanned this gene boundary. Details for all barcode primers used and annealing conditions are given in Table C of S1 File.

Thrips are very small insects and total genomic DNA yield was generally low. To overcome possible problems of insufficient template for use with non-specific barcode primers, three strategies were used. One strategy was to employ a Nested PCR protocol. The second strategy was to increase overall yield with whole genome amplification using the GenomiPhi V2 kit (GE Healthcare, Piscataway, NJ). The third strategy was the use of specific primers to amplify a smaller fragment of the COI gene [30] (Table C of S1 File). Additionally, evidence was found of both heteroplasmy and pseudogenes so sequences were often validated by more than one primer pair, especially when they represented unique/novel haplotypes or when evidence of pseudogenes was found. Polymerase chain reactions were run using either GoTaq (Promega, Madison, WI) or Supermix (Thermo Fisher, Waltham, MA) kits, PCR products were visualized on a 1.5% agarose gel via electrophoresis, cleaned using Montage (EMD Millipore, Danvers, MA) or Nucleospin (Machery-Nagel, Bethlehem, PA) clean-up kits, and directly sequenced bi-directionally using a BigDye Terminator cycle sequencing kit and an 3730XL DNA sequencer (both Thermo Fisher). Sequences were contiged, traces inspected, and base calls edited using Sequencher v4.8 (Gene Codes, Ann Arbor, MI). Sequences were compared to those already in the NCBI nr nucleotide database using BLAST [31].

### Nuclear Genetic Loci

Two next-generation sequencing datasets were used for comparative genomics to identify homologous nuclear loci with a relatively high degree of divergence among the South Asia 1 and East Asia 1 cryptic species (see results). DNA was extracted from a single individual from Japan representing the East Asia 1 cryptic species. First the thrips was incubated at 56°C for four hours in 75uL lysis buffer and 10uL proteinase K (Qiagen Inc., Valencia, CA). Then 75uL each of phenol then chloroform (both Sigma-Aldrich) were added, centrifuging for 10 min at 13,200rpm and 4°C following the addition of each chemical and removing the aqueous layer after each centrifugation step. DNA was then precipitated overnight at 4°C with isopropanol (2/5 volume) and sodium acetate (1/10 volume), centrifuged for 20 minutes, and the DNA pellet washed with 100μl iced 100% ethanol, dried at room temperature, and re-suspended in 10μl nuclease free water. The details for DNA extraction from a second sample from Florida representing the South Asia 1 cryptic species have been reported previously [32]. The Ion Torrent system (Thermo Fisher) was used for next-generation sequencing with these two samples and details including reagent kits, hardware, and preliminary analyses to create contigs from raw sequencing reads are provided in Dickey *et al*. [32] and were consistent for both samples. Comparative genomics was done via bi-directional best hit analysis with a threshold eValue of 10^-50^. 199 contigs from each dataset were bidirectional best matches given this criterion (not shown) and from these, seven loci were selected for sequencing in support of phylogenetic reconstruction (Table D of S1 File). These were aligned in Geneious v6 (Biomatters, Auckland, New Zealand) for primer design conducted in Primer3 [33]. These seven loci + 28S-D2 [34] were sequenced for 17 individuals from 10 countries representing six cryptic species within the complex (Table A of S1 File). DNA for these samples was subjected to whole genome amplification (see *Barcoding*) to maximize the template available for PCR reactions. Introns were located using the Augustus web server [35] with the *Acyrthosiphon pisum* training set.

### Cryptic Species Delimitation

Unique COI haplotypes containing at least the first 640 nucleotides of the 655 folmer barcode region [26] and no fully ambiguous base calls were shortened to the minimum length and all pairwise genetic distances were calculated using PAUP* [36] using the TIM3+I model to account for state frequency and site rate variation. These distances were used in histogram analysis after Dinsdale *et al*. [37] to search for natural breaks in the genetic distance data. This analysis included all unique sequences from this study and 38 unique sequences downloaded from GenBank including four putative outgroups (Table B of S1 File). However one GenBank sample exhibiting many pairwise distances that bordered a natural break was removed before phylogenetic analyses due to an elevated number of transversion substitutions from the most similar consensus sequence suggesting it could be a pseudogene [38]. A second singleton sequence also exhibited many pairwise distances that bordered a natural break in the data but it had an expected transition/transversion ratio. This sequence also exhibited conflicting gene phylogenies among eight nuclear and one mitochondrial locus suggesting possible incomplete lineage sorting. To test the hypothesis that this was a unique species (provisionally designated South Asia 3, see results), species delimitation analysis was conducted based on the multi-species coalescent implemented in BPP [39–40]. All available gene sequences from all members of three putative terminal taxa in the South Asia clade (South Asia 1, 2, and 3 see results) were included with a maximum of 12, 3, and 1 sequences per species at each of the nine loci. The guide tree was the consensus of all individual gene trees: South Asia 1, (South Asia 2, South Asia 3) (see *Phylogenetic Inference*). The analysis was run for 100,000 generations sampling every 2 with 20% discarded as burn-in. Algorithm 1 was used with gamma priors (1, 10) on the ancestral population size (*θ*_*S*_), gamma priors (2, 2000) on the root age (*τ*_*0*_) and Dirichlet priors for all other values of *τ*. These priors have been shown to be the most conservative for delimiting speciation events [41]. Four independent runs were conducted to ensure that all three possible starting trees were assigned as the prior at least once and fine-tuning parameters were adjusted to ensure recommended proposal acceptance rates between 0.3 and 0.7. Support for the putative species identified by histogram analysis was also evaluated based on monophyly and posterior support for a clade in the COI gene tree (details in *Phylogenetic Inference*).

### Phylogenetic Inference

Each gene tree was inferred separately using all unique sequences. The species tree was inferred using the concatenated nine locus consensus sequence of each putative species. The amount of missing data for any terminal in any analysis did not exceed 83%. The gene tree with the extended COI barcode included all unique sequences from this study plus 38 unique sequences downloaded from GenBank including puataive outgroups (Table B of S1 File). Due to the high number of putative species represented by a single individual, this analysis was also conducted with putative species represented by a single individual removed. The 28S-D2 gene tree contained all unique sequences from this study and five unique sequences from GenBank including putative outgroups. A two gene concatenated phylogeny (28S-D2 and COI) was also constructed with only sequenced individuals that were unique for one or both genes. Gene trees for the remaining seven loci contained only unique sequences from this study. Saturation was tested using Xia’s Test [42] in DAMBE [43] and detected for COI third codon position sites (see results). Because of this, analyses containing COI were done both with and without third position sites and with RY coding. Third position sites were removed prior to species tree inference to ensure that deep nodes in the phylogeny were recovered without the effect of this saturation. Alignments were created using Clustal X [44] and manually in Mesquite v2.75 [45]. Phylogenetic analyses using Bayesian inference were conducted using MRBAYES v3.2 [46] using two independent runs of 4–12 chains. Convergence of the posterior was assessed using the average standard deviation of split frequencies between runs (<0.01), and an effective sample size of each estimated parameter (>200) as determined in Tracer v1.6 [47]. Samples prior to posterior convergence were discarded as burn-in. Partitioning datasets was done as follows: first the number of parsimony informative sites was determined for noncoding portions and each codon position of each gene using PAUP*. Second, codon positions for each gene were consolidated if they had fewer than three parsimony informative sites. Third, the optimal partitioning strategy was determined for each gene using PartitionFinder v1.1 [48]. For multi-gene analyses, PartitionFinder was run a second time treating previously identified partitions for each gene as blocks (however nuclear and mitochondrial derived blocks were restricted from being united in a partition). Fourth, the partitioning strategy was evaluated against the unpartitioned dataset using Bayes factors calculated using the stepping-stone method [49] in MrBayes. Bayes factors were calculated using marginal likelihoods according to the formula: BF = 2 × (ln L_1_—ln L_0_) + (P_1_—P_0_) × ln (0.01) [50] where P is the number of parameters and L is the marginal likelihood of the null (unpartitioned) and alternative (partitioned) analysis. The best model of sequence evolution for un-partitioned analyses was determined using the Bayesian information criterion in ModelTest [51]. The sumtrees script in Dendropy v3.12 [52], Figtree v1.4 [53] and Mesquite were used for summarizing, viewing and manipulating trees. Trees were treated as unrooted during all analyses and the root was determined by examining the locations of putative outgroup taxa at the end of species tree inference. Four taxa were used as putative outgroups: *S*. *inermis*, *S*. *aff*. *dobroskyi*, *S*. *oligochaetus*, and the recently described *S*. *aff*. *dorsalis* [54].

### Population Genetic and Phylogeographic Analyses

The abbreviated barcode was used to estimate the following population genetic parameters for populations with n≥4: haplotype diversity (HD), nucleotide diversity (*π*), and root age (τ) using DNAsp v5 [55]. Exact tests of differentiation among populations of the same cryptic species (n≥7) were conducted in Arlequin v3.5 [56] using default settings and populations were consolidated for additional analyses according to the results. Analysis of molecular variance (AMOVA) was used to assess geographic structure among Indian populations using 16,000 permutations under the Tamura & Nei model of sequence evolution [57] in Arlequin. The possibility of rapid historical demographic expansion for *S*. *dorsalis* populations was investigated using the mismatch distribution [58] as well as several statistics under the expectations of rapid population growth: raggedness [59], R2 [60], Fu and Li’s F* and D* [61], Tajima’s D [62] and Fu’s F_S_ [63]. Arlequin was used to conduct neutrality tests (Tajima’s D and Fu’s F_S_) using 16,000 simulated samples and goodness-of-fit tests under the mismatch distribution using 16,000 bootstrap replicates. DNAsp was used for all other tests using 16,000 coalescent simulations. Cases of demographic expansion were dated using τ, 13.5 generations per year [64], and a 6.5% per-million-years pair-wise divergence rate inferred between head and body lice [65] implemented in the online mismatch calculator [66]. Thrips have significantly higher nucleotide substitution rates in their mitochondrial genomes than do flies: Phthiraptera ~ Thysanoptera > Hymenoptera > Other Holometabola [67] justifying the use of an elevated substitution rate for COI here rather than the 2.3% rate reported for drosophila [68]. In addition, intraspecific divergence rates are also argued to be elevated relative to interspecific divergence rates [69].

### New York

The population from New York, USA (Table A of S1 File) arrived during the final stage of manuscript preparation. Barcodes from this population were thus excluded from many analyses however a single locus phylogenetic analysis was conducted to include these haplotypes. This phylogenetic analysis used only East Asia 1 cryptic species haplotypes with East Asia 3 to root the phylogeny (see results). A single nuclear locus, htpG, was also used to confirm the barcode identification.

## Results

100 unique DNA barcode haplotypes were sequenced from 494 *S*. *dorsalis* and four *S*. *aff*. *dorsalis* individuals. The slide-mounted voucher specimens were high quality and observable characters of each were consistent with *S*. *dorsalis*. Heteroplasmy was found in two samples from Israel and pseudogenes were rare and often resulted from reduced temperature PCR (Table C of S1 File). Nuclear loci generally had <1% polymorphism/ambiguity, however for two genes, POLD1 and HZF the East Asia 2 sample had higher ambiguity (3% and 14% respectively) (Table 1). These sequences were retained for phylogenetic analysis. All sequences have been deposited in GenBank (Table A of S1 File).

**Table 1:** Sequencing results for eight nuclear loci.

Gene	Unique sequences	Length				Introns	Missing data^1^ ^,^ ^2^	Missing taxa^2^	Ambiguity/ polymorphism^1^
		Coding	Non-coding	Total	Aligned				
CAD	10	645	0	645	645	0	none	none	0%
DCR1	10	257	167–189	424–446	446	2	none	none	0–0.2%
POLD1	9	280	187–204	467–484	485	2	none	none	0–3%
TIF31	7	536	0	536	536	0	none	none	0%
htpG	8	229	0	229	229	0	none	E Asia 2	0%
ESRP1_2	7	337	80–83	417–420	420	1	21% from *S*. *aff*. *dorsalis*	E Asia 2	0%
HZF	8	223	76–82	299–305	305	1	40% from E Asia 2	none	0–14%
28S-D2	14*	-	-	537–538	539	-	none	E Asia 2	0–0.2%

^1^Missing data and ambiguity/polymorphism were slightly higher than zero for some samples used for species delimiting with the multi-species coalescent (South Asia 1 and 2 cryptic species). These parameters were minimized for phylogenetic inference by selecting the most complete unique sequence for each taxa.

^2^From among six cryptic species sampled (S1 Table).

*Includes nine sequences from Hoddle et al. [7] (Table B of S1 File).

### Cryptic Species Delimitation

**Fig 2:**
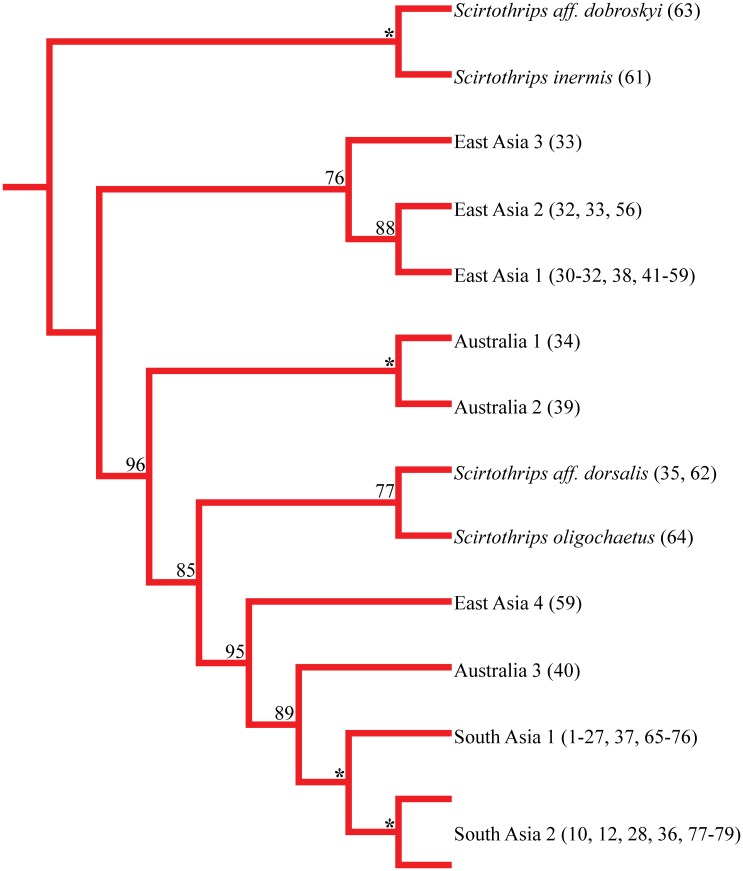
Bayesian species-level phylogeny of the *Scirtothrips dorsalis* complex. Based on the consensus sequences of nine concatenated loci for each species and including DNA sequences from this study (Table A of S1 File) and those mined from GenBank (Table B of S1 File). Support subtending each node is the posterior clade frequency out of 100. * denotes frequency in >99.9% of trees.

**Fig 3:**
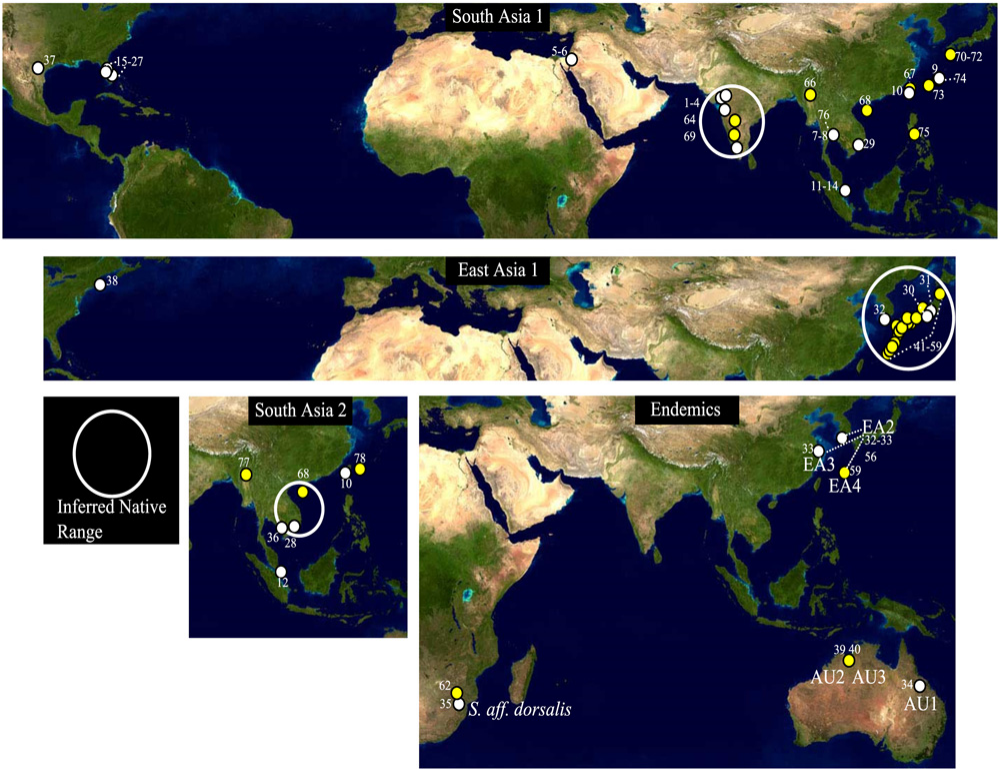
Ranges of species within the *Scirtothrips dorsalis* complex. AU1- Australia 1, AU2- Australia 2, AU3- Australia 3, EA2- East Asia 2, EA3- East Asia 3, EA4- East Asia 4. Includes haplotypes detected in this study (white, Table A of S1 File) and mined from GenBank (yellow, Table B of S1 File). The earth global view used in this figure consists of MODIS satellite data for the NASA Blue Marble 2002 project. These images are freely available to educators, scientists, museums, and the public.

Major breaks in pairwise genetic distances occurred at 2.7% and 9.7% sequence divergence. Provisionally assigning species status according to the 2.7% cut-off gave twelve species within the complex (ten cryptic, *S*. *oligochaetus* and *S*. *aff*. *dorsalis*). In single locus DNA barcode phylogenetic analyses, provisional species were not always recovered as monophyletic with strong support (Table 2) with problems occurring within well-supported clades corresponding to the 9.7% sequence divergence breakpoint. Cryptic species have been assigned names based on their inferred native range (Figs 2 and 3). Within the South Asia 1–3 clade (S2 Fig), the multi-species coalescent resolved South Asia 1 as distinct (posterior probability of speciation = 100%) but South Asia 2 and 3 were consolidated as a single species (posterior probability of speciation = 25%) reducing the number of species in the complex to eleven. The singleton representative of the unsubstantiated South Asia 3 cryptic species grouped with South Asia 1 in COI (S1 Fig) and DCR1 (S2 Fig) gene trees but grouped with South Asia 2 in the remaining 7 gene trees (S2 Fig). Within the East Asia 1–3 clade (S1 Fig), the three putative species were always recovered as monophyletic when the third codon position was included though posterior clade support subtending the East Asia 1 species was often low (Table 2). Most species in the complex are restricted to a small geographic region while South Asia 1 and 2 and East Asia 1 have more extensive geographic ranges (Fig 3).

**Table 2:** Single locus monophyly tests of select *Scirtothrips dorsalis* cryptic species.

Species	Phylogenetic analysis^1^	Monophyly?	Posterior clade probability
East Asia 1	1	Yes	82
	2	Yes	61
	3	Yes	51
East Asia 2	1	Yes	100
	2	Yes	100
	3	Yes	85
South Asia 1	1	Yes	72
	2	No	-
	3	No	-
South Asia 2	1	No	-
	2	Yes	100
	3	No	-

^1^1-All unique barcodes, 2-No singleton species representatives, 3-No third codon sites.

### Phylogenetic Inference

Bayes factors always favored partitioning datasets (Table 3). The third codon position COI saturation index of the data was not significantly different from the critical value indicating substantial saturation and justifying their removal (Iss = 0.661, Iss.c = 0.693, P = 0.36). Bayesian analyses converged and adequately sampled the posterior. The nine locus phylogeny showed eleven species within the *S*. *dorsalis* complex with *S*. *aff*. *dobroskyi* and *S*. *inermis* rooting the tree and *S*. *oligochaetus* and *S*. *aff*. *dorsalis* rendering morphological *S*. *dorsalis* polyphyletic (Fig 2). Support for nodes varied but each split occurred in >75% of trees. Support for the East Asia 1–3 clade was 76% in the species tree but was 100% in the COI gene tree (S1 Fig) suggesting some phylogenetic information was lost for this shallower node by removing third codon position sites. Support for the *S*. *aff*. *dorsalis S*. *oligochaetus* clade was 77% and this clade only emerged with modest support when the COI third codon position was removed and combined with the 28S-D2 locus (S1 Fig). The position of the Australia 1–2 clade and *S*. *aff*. *dorsalis S*. *oligochaetus* clade was reversed in the combined 28S-D2 barcode analysis when third codon positions were removed (S1 Fig) but this split in the species tree is present and has strong support in each of the seven remaining nuclear gene trees (range 74–100%, S2 Fig). Support for both the South Asia 1–2 and Australia 1–2 clades exceeded 99.9%. Monophyly of the ingroup was strongly supported by the 28S-D2 gene tree but was not supported by the COI gene tree, even when third codon position sites were removed (S1 Fig). The East Asia 1–3 clade was recovered as the most basal within the ingroup.

**Table 3:** Bayes factors favor partitioned over unpartitioned phylogenetic analyses.

Analysis	Partitions	Bayes factor^1^
CAD	2	22
DCR1	3	86
POLD1	2	85
TIF31	2	51
HZF	2	10
COI (no third codon sites)	2	287
ESRP1_2	2	50
Species tree	6	503

^1^A Bayes factor of at least 10 is interpreted as significant favoring the more complex partitioned model after Kass & Raftery [70].

**Fig 4:**
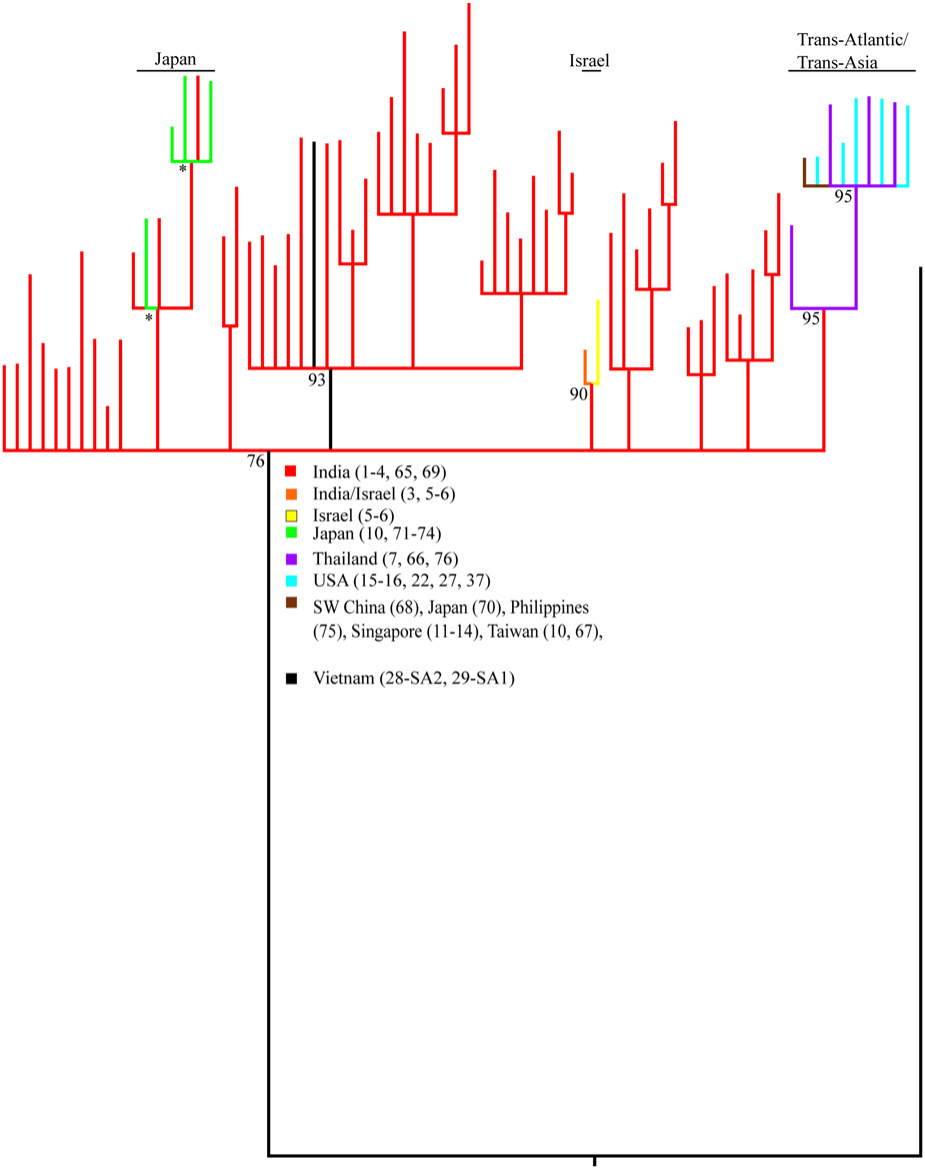
South Asia 1 cryptic species portion of the *Scirtothrips dorsalis* COI gene tree (75% consensus) showing multiple invasive maternal lineages now present in Israel (orange/yellow), Japan (green), and South Asia, East Asia, and North America (purple, blue, brown). Support subtending each node is the posterior clade frequency out of 100. * denotes frequency in >99.9% of trees. A single member of the South Asia 2 cryptic species roots the tree. Includes DNA sequences from this study (Table A of S1 File) and those mined from GenBank (Table B of S1 File).

At least three independent South Asia 1 haplotype groups have invaded different parts of the world (Fig 4). One lineage contains haplotypes detected in both India and Japan. A second lineage contains haplotypes detected in India and Israel, including one shared haplotype. A third lineage contains six haplotypes in the US and five in Thailand (one shared). All lineages in the phylogeny arise from within 52 unique Indian haplotypes. A single common haplotype found in this study and reported in GenBank has been detected throughout South Asia, East Asia, and the Southern United States. This common haplotype was found in 310 (63%) samples in this study and in >97% of all South Asia 1 samples from the US, Singapore, Thailand, and Taiwan. East Asia 1 has invaded New York, USA (Fig 3).

### Population Genetic and Phylogeographic Analyses

**Fig 5:**
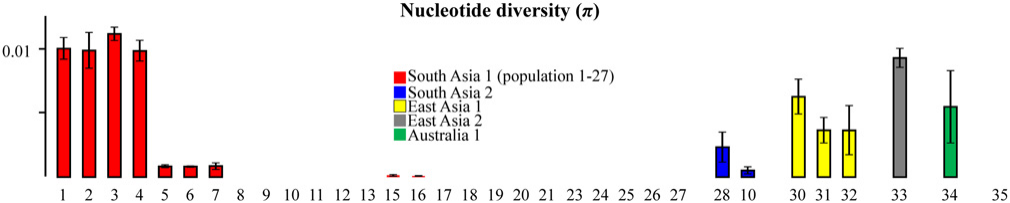
Nucleotide diversity (*π*) for 33 populations of 6 species within the *Scirtothrips dorsalis* species complex (Table A of S1 File). Numbered populations correspond to Table 4. Population 35 is *S*. *aff*. *dorsalis*.

Populations could be generally described as either having low nucleotide diversity (*π*) (few haplotypes separated by a single mutation) or moderate-high *π* (few-to-many haplotypes separated by multiple mutations) (Fig 5, Table 4). For South Asia 1 populations in India, haplotype diversity approached 1 with 52 haplotypes recovered from 70 individuals and *π* ~0.01 (populations 1–4). *π* was moderate to high within East Asia 1, East Asia 2, and Australia 1 populations but was low for *S*. *aff*. *dorsalis* (Fig 5). South Asia 2 contained two populations; one with moderate *π* (Vietnam, population 28) and one with low *π* (Taiwan, population 10) (Fig 5). Populations 10, 12, 32 and 33 contained more than one cryptic species (syntopy) (Table A of S1 Fig).

**Table 4:** Population genetics parameters and tests.

Code	Location/*Host*	n	%C	π	HD	Τ	rg	R2	F*	D*	D	Fs	ND	E_D_	E_S_
1	Maharashtra	10	0	0.011	1.00	6.87	0.06	**0.10**	-0.9	-0.9	-0.74	**-4.7**	-	-	-
2	Tamil Nadu	20	0	0.010	0.99	6.31	0.03	0.09	-1.3	-1.2	-0.89	**-10.7**	-	-	-
3	Madhya Pradesh	27	0	0.011	0.97	7.13	0.03	0.08	-1.9	-1.8	-1.04	**-11.4**	-	-	-
4	Gujarat	13	0	0.010	0.90	4.47	0.08	0.15	0.2	0.1	0.11	-0.1	-	-	-
	India		0	0.011	0.97	6.74	0.02	0.05	**-3.3**	**-3.6**	-1.47	**-49.2**	0.2	**0.002**	**0.015**
5	Israel 2009	13	0	0.001	0.54	0.54	0.30	*0*.*27*	1.0	0.7	1.46	1.2	-	-	-
6	Israel 2008	12	0	0.001	0.53	0.53	0.28	0.27	1.0	0.8	1.38	1.2	-	-	-
	Israel		0	0.001	0.51	0.51	0.26	*0*.*26*	1.0	0.6	1.56	1.6	1.0	**0.040**	**0.006**
7	Thailand Field	10	70	0.001	0.51	0.56	0.18	0.17	-0.4	-0.3	-0.69	-0.6	-	-	-
8	Thailand Ornamentals^1^	29	100	-	0	-	-	-	-	-	-	-	-	-	-
9	Okinawa	4	0	-	0	-	-	-	-	-	-	-	-	-	-
10	Taiwan (South Asia 1)	8	100	-	0	-	-	-	-	-	-	-	-	-	-
11	Singapore *Rosa*	32	100	-	0	-	-	-	-	-	-	-	-	-	-
12	Singapore *Chrysanthemum*	9	100	-	0	-	-	-	-	-	-	-	-	-	-
13	Singapore *Capsicum*	23	100	-	0	-	-	-	-	-	-	-	-	-	-
15	Florida *Rosa*	22	95	1E-04	0.09	0.09	*0*.*68*	*0*.*21*	-1.7	-1.6	-1.16	-1.0	-	-	-
16	Florida *Raphiolepis*	34	97	9E-05	0.06	0.06	*0*.*78*	0.17	-1.8	-1.7	-1.14	-1.3	-	-	-
17	Florida *Viburnum*	5	100	-	0	-	-	-	-	-	-	-	-	-	-
18	Florida *Gossypium*	11	100	-	0	-	-	-	-	-	-	-	-	-	-
19	Florida *Capsicum*	11	100	-	0	-	-	-	-	-	-	-	-	-	-
20	Florida *Capsicum* var Chilly	16	100	-	0	-	-	-	-	-	-	-	-	-	-
21	Florida *Ocimum*	23	100	-	0	-	-	-	-	-	-	-	-	-	-
23	Florida *Citrus* Lake Alfred	11	100	-	0	-	-	-	-	-	-	-	-	-	-
24	Florida *Fragaria*	9	100	-	0	-	-	-	-	-	-	-	-	-	-
25	Florida *Vaccinium*	44	100	-	0	-	-	-	-	-	-	-	-	-	-
26	Florida *Shefflera*	11	100	-	0	-	-	-	-	-	-	-	-	-	-
27	Florida *Citrus* Fort Pierce	5	100	-	0	-	-	-	-	-	-	-	-	-	-
	Invasive^2^	293	99	2E-05	0.01	0.01	*0*.*95*	0.04	-3.1	-3.2	-1.24	-5.7	0.6	0.058	0.980
28	Vietnam	4	0	0.002	0.50	0.41	0.75	*0*.*43*	-0.7	-0.8	-0.75	1.7	-	-	-
10	Taiwan (South Asia 2)	6	0	0.001	0.33	0.33	0.22	*0*.*37*	-1.0	-1.0	-0.93	0.0	-	-	-
30	Shizuoka	15	0	0.006	0.79	1.21	0.09	0.15	-0.9	-0.9	-0.53	1.1	-	0.400	0.527
31	Ibaraki	14	0	0.004	0.63	0.22	0.19	0.20	1.3	1.3	0.86	3.1	-	0.203	0.169
32	Jeju	6	0	0.004	0.60	0	0.20	0.31	-1.5	-1.4	-1.39	1.3	-	-	-
33	Hangzhou	20	0	0.009	0.80	2.60	0.14	0.13	-0.8	-1.0	-0.85	1.3	-	**0.027**	0.267
34	Australia	4	0	0.006	0.50	0.15	0.75	*0*.*43*	-0.8	-0.8	-0.82	3.3	-	-	-
35	*S*. *aff*. *dorsalis*	4	0	-	0	-	-	-	-	-		-	-	-	-

-Abbreviations: n-sample size, %C-frequency of common invasive haplotype.

-Descriptive statistics: π-nucleotide diversity, HD-haplotype diversity, τ-the date of population growth in mutational time under the expansion model, rg-raggedness, F*-Fu&Li’s F*, D*-Fu&Li’s D*, D-Tajima’s D, Fs-Fu’s Fs.

-Statistical tests: ND-probability of non-differentiation (used to justify combining India, Israel, and select invasive populations as panmictic), E_D_-test of demographic expansion p-value, E_S_-test of spatial expansion p-value.

-Descriptive statistics could not be calculated for populations with HD = 0.

-Expansion tests were conducted for panmictic populations and populations where n>7.

-Significance is assessed under a two-tailed test for all test statistics and ND and a one-tailed test for E_D_ and E_S_.

-Bold denotes significance favoring expansion while italics denote significance rejecting an expansion model.

^1^Farris *et al*. [12];

^2^The invasive panmictic population includes all populations with a sample size >7 and a common invasive haplotype frequency >90%.

There was no geographic structure detected among Indian populations of South Asia 1 (AMOVA among sites 0.69, P = 0.28, exact test P = 0.19). Treating these as a single population indicated a history of rapid population growth (P = 0.002) and/or spatial expansion (P = 0.0145) (Table 4). These expansion models were corroborated by other tests, Fu’s F_S_ (-49.2, P<10^-5^), Fu and Li’s F* (-3.329, P = 0.007), Fu and Li’s D* (-3.614, P = 0.005) and low values of raggedness (0.021), R2 (0.051), and Tajima’s D (-1.469) test statistics. Israel (populations 5–6 combined, South Asia 1) and Hangzhou, China (population 33, East Asia 2) populations also had significant demographic expansion signals under the simulation test in Arlequin (Table 4) but had tests and/or test statistics in conflict with a population growth model; a significantly high R2 in Israel (0.269, P<10^-5^), and high raggedness, though not significant, in Hangzhou, China (0.14, p-value 0.19). The population growth model for East Asia 2 had no corroborating support (Table 4). The rapid population growth event of South Asia 1 in India was dated to ~160,000 years before present.

## Discussion

### Utility of COI

Though the universal application of the 3% divergence barcode cut-off for delimiting species [71] has been discredited in many specific instances [72–73], it has also been independently found as a naturally occurring breakpoint in several taxon specific barcode libraries [37,71,74]. COI was useful for delimiting species in the case of the *S*. *dorsalis* complex, but was not a panacea and when used alone was misleading in one instance. Only by applying the multi-species coalescent was the putative South Asia 3 cryptic species consolidated with South Asia 2.

All Mitochondrial pseudogenes in the nucleus (Numts) clustered together phylogenetically due to their close similarity to the globally common South Asia 1 haplotype (not shown). Definitive numts found in this study, based on in-frame stop codons, were found in both the South Asia 1 and East Asia 2 cryptic species. If the numts had formed a distinct clade as found for *Hylaeus* bees [37], it would have confirmed the prediction that numts would cause an overestimate of species diversity [75]. But our results indicate that in some cases the opposite could be true. Numts could lead to an underestimate of species diversity and would have in the case of the *S*. *dorsalis* complex with East Asia 2 incorrectly identified as invasive South Asia 1.

The elevated nucleotide diversity found in the native ranges of multiple members of the *S*. *dorsalis* complex (Table 4, Fig 5) may be the result of an elevated rate of substitution in mitochondrial genes of thrips [67]. This feature of COI proved useful for discriminating among high and low diversity populations (Fig 5) but precluded detection of spatially explicit population structure and unambiguous assignment of invasive lineages to a single high diversity population as being its origin. This was true for both South Asia 1 (Table 4, Fig 4) and East Asia 1 (not shown) invasive haplotypes. Primer failure may also be partially blamed on primer/template mismatch exaggerated by an increased third position substitution rate. In addition to the barcode locus, lower diversity nuclear markers such as provided in Table D of S1 File may prove useful for identifying members of the *S*. *dorsalis* complex.

### Scirtothrips dorsalis Species Complex Phylogeny

Morphological *S*. *dorsalis* is rendered polyphyletic by *S*. *oligochaetus* and *S*. *aff*. *dorsalis* as previously reported [7]. The eleven species within phylogenetic *S*. *dorsalis* (nine morphologically indistinguishable members plus *S*. *aff*. *dorsalis* and *S*. *oligochaetus*) should be considered a minimum estimate of the total number of species in the complex (Fig 2). With greater geographic and host sampling, especially from wild hosts, this number is likely to rise as with other cryptic species complexes containing pest members [21,76]. There is a general geographic pattern in the phylogeny with South Asia, East Asia, and Australian species often resolved as distinct clades within the tree and East Asia 4 and Australia 3 being exceptions. Missing data was somewhat problematic but surmountable. *S*. *oligochaetus* was the most rogue taxa within the phylogeny, its position in the tree not emerging as presented until combining 28S-D2 with COI and removing COI third codon position sites. As such, *S*. *oligochaetus* would benefit the most from increased gene sampling.

Toda *et al*. [10] described two ‘strains’ of *S*. *dorsalis* in Japan, however these two strains correspond to four cryptic species in the framework presented here. The majority of their Strain C corresponds to South Asia 1 while the haplotype designated SdC06 (GenBank accession AB818023) belongs to South Asia 2. In addition, the majority of their Strain YT corresponds to East Asia 1 while haplotype SdYT21 (AB818048) belongs to East Asia 2. Also consistent with our results, Rebijith *et al*. [11] resolved as distinct South Asia 1 and 2 designating them Group II and Group I respectively. Future molecular characterization in this system and development of molecular diagnostics for identification should benefit from the expansion of the barcode library and the phylogenetic framework presented here.

Certainly more morphological characters are needed for the *S*. *dorsalis* species complex, but this is true of Thysanoptera in general. Given the relatively few characters available and the often high level of intraspecific morphological variation, relying solely on morphology to identify *Scirtothrips* spp. is discouraged [77]. The permanent vouchers created as part of studies such as this one and [7], will be invaluable for researchers to subsequently examine in light of results gained with molecular data.

### Host Use and Tospovirus Transmission

*S*. *dorsalis* is reported to be highly polyphagous [78] feeding on more than 100 species in 40 different plant families. However, revising as a cryptic species complex has the potential for reducing the host range of some members. When considering barcode data some species in the complex remain polyphagous (Table 5) and this should be updated with accompanying molecular identification. In Japan, where *S*. *dorsalis* (East Asia 1) has long been reported as a pest of grape and citrus, South Asia 1 has only recently become a pest in chilli and mango where it is adventive [10]. In contrast, other species a in the complex appear monophagous (Table 5). *S*. *aff*. *dorsalis* is known only from *Ricinus sp*. in South Africa and has been sampled exclusively from this host for the past 30 years [6,54].

**Table 5:** Important biological characteristics of species within the *S*. *dorsalis* complex.

Species	Host range^1^(number of host genera)	Pest status^2^	Invasion potential^3^
South Asia 1	high(20)	pest	high
South Asia 2	high(10)	pest	moderate
East Asia 1	high(7)	pest	moderate
East Asia 2	moderate(3)	pest	low
East Asia 3	low(1)	pest	low
East Asia 4	low(1)	benign	low
Australia 1	low(1)	pest	low
Australia 2	low(1)	benign	low
Australia 3	low(1)	benign	low
*S*. *aff*. *dorsalis*	low(1)	benign	low
*S*. *oligochaetus*	high(7)	pest	low

^1^Host range is determined here based on DNA barcode records (Tables A-B of S1 File) or from [5] for *S*. *oligochaetus* (Table B of S1 File) and is likely higher for many species within the complex.

^2^Pest status is determined here based on detection on at least one commercial host.

^3^Invasion potential is determined based on a “history of invasion” criterion [23]. This is inferred based on range maps (Fig 3) and the presence of low diversity (Fig 5) or newly detected populations at the periphery of ranges.

*S*. *dorsalis* is one of only 14 species of Thysanoptera known to transmit tospoviruses [79]. G and L cryptic species of *F*. *occidentalis* differ in their vector efficiency [20] and this may also be the case for the members of the *S*. *dorsalis* complex. South Asia 1 is likely the species implicated in tospovirus transmission as it is present in India, Taiwan, and Thailand where vectoring has been documented [80–83]. In Taiwan and Thailand where South Asia 1 and 2 co-occur, the vectoring ability of these two species should be compared.

### Endemism and Invasion

Climatological modeling presumed *S*. *dorsalis* would not permanently colonize areas in the U.S. with sustained overwintering temperatures below -4°C [84], but see [85]. However, exclusion zones based on temperature may be different for different members of the *S*. *dorsalis* species complex. For example, East Asia 1 appears to have overwintered during 2013/2014 in New York, USA, in a nursery hoop house under plastic cover, based on very early season damage to hydrangea (Dan Gilrein, *Personal communication*). This location lies well outside the proposed minimum temperature exclusion area, and the overwintering would have occurred during a particularly cold winter. East Asia 1 may have a greater cold tolerance than tropical members of the complex due to its native range extending into temperate portions of Japan [10] and this should be considered when determining the range expansion potential of each member of the *S*. *dorsalis* complex.

Endemism and an organism’s native range can be inferred based on two criteria using genetic data: 1) If a species is found in only one location, it is inferred to be endemic and native to that place. 2) If a species has a high degree of genetic diversity in one particular region and a lower degree of genetic diversity elsewhere it can be inferred that that species is native to the area possessing high genetic diversity and recently invasive in a region possessing low genetic diversity because of an expected bottleneck upon invasion [14]. This assumes a constant mutation rate for the locus. Given the first criteria, it appears that seven species are endemic with restricted ranges (Fig 3). In addition, *S*. *oligochaetus* has been reported from both India and Africa [5]. These eight species may have low invasion risk (Table 5) under the “history of invasion” criterion [23] but future risk evaluations should also consider the pest status, host range, and virus transmission history of these species in their native range.

South Asia 1 is highly invasive globally (Fig 3). Indian populations had the highest genetic diversity with 52 haplotypes detected and high π (Populations 1–4 in Fig 5). We thus consider India to be part of the native range of South Asia 1 given criteria 2. High priority should be given to determining the full extent of the native range of South Asia 1 (Pakistan, Bangladesh, Myanmar, etc.) through comprehensive geographic and host sampling, especially around the Bay of Bengal. Thailand, though possessing moderate haplotype diversity in one field population (Table 4), has low nucleotide diversity (Populations 7–8 in Fig 5) consistent with South Asia 1 being invasive in Thailand. This should also be confirmed through more extensive geographic and host sampling. The possibility that the South Asia 1 species is native to Thailand cannot be ruled out at this time but genetic diversity under criteria 2 suggests otherwise. Even if not native, Thailand may represent a bridgehead population [14] preceding and giving rise to the more widespread and recent global invasion since the trans-Atlantic/trans-Asia invasive haplotype lineage contains haplotypes unique to Thailand (Fig 4), including one predicted by the phylogeny to be ancestral. In addition to this globally common haplotype lineage, at least two separate South Asia 1 haplotype groups have independently invaded Japan and Israel. A fourth possible invasion represented by a single thrips collected from Mango in Vietnam (Table A of S1 File, Fig 4) should also be confirmed by population-scale sampling. The multiple invasive maternal lineages within South Asia 1 were not able to be traced back to a specific region within the native range due to a lack of geographic structure in the genetic data. Despite this, all invasive lineages of South Asia 1 are placed phylogenetically among Indian haplotypes (Fig 4) and likely originated in India or some adjacent region not sampled.

South Asia 2 is represented by nine haplotypes from this study and three haplotypes from GenBank for a total of 12 unique haplotypes. The highest number of haplotypes present at any location is five from Unicorn Island, Vietnam, though it is unknown whether multiple hosts were sampled at this location or whether these represent a single interbreeding population (Table A of S1 File). None-the-less, even when treating the four least divergent samples as a single population, *π* is moderate (Population 26 in Fig 5). An additional 4 haplotypes are reported from Hainan Island, China (Table B of S1 File). These two sites together account for 75% of the haplotype diversity documented to date in this cryptic species leading us to provisionally assign these two sites as part of the native range (Fig 3). However, sampling and detection is limited and further population-scale sampling is needed in this region to distinguish between high and low genetic diversity populations. The presence of a low diversity population of this species in Taiwan (Population 27 in Fig 5) coupled with the recent detection of one of these Taiwanese haplotypes on mango in Japan (GenBank accession AB818023 in [10]) suggests that South Asia 2 may be invasive in these two locations.

East Asia 1 has relatively high π in its native range, which includes portions of Japan and South Korea (Populations 28–30 in Fig 5, Fig 3). In addition to our study, 38 East Asia 1 haplotypes were found by Toda *et al*. [10] throughout Japan so we consider Japan to be part of the native range. This species has a relatively high host-range (Table 5) and a history as a documented pest in Japan going back 55 years [86]. This species should now be considered an invasion threat as it is present in New York, USA (Fig 3). *S*. *dorsalis* was first identified morphologically in New York in 2012 with damage to hydrangeas noted in late 2011 (Dan Gilrein, *Personal communication*). This species may also be a transplant risk within the US with less strict climatic requirements compared to tropical members of the complex. This species has been documented from hydrangeas and azaleas in New York but not on roses planted nearby (Dan Gilrein, *Personal communication*). Rose is a preferred host of South Asia 1 in Florida and Texas.

More than one member of the complex is invasive (Fig 3, Table 5) and will need to be discriminated using molecular methods by organizations implementing national pest quarantine and exclusion tasks to limit spread and introduction to new areas. The invasive members of the complex have moderate to high host ranges (Table 5) demonstrating their potential to become agricultural pests where introduced. The reduced range of South Asia 2 relative to South Asia 1 (Fig 3) suggests an earlier stage of invasion that can still be limited and/or slowed. In addition, East Asia 1 likely invaded New York, USA within the past three years and its identity was confirmed with DNA barcoding in August, 2014. This invasion may also be in its infancy. The molecular tools in this study (Tables C-D of S1 File) are useful for diagnostics when a DNA sequencer is available and should be used when assigning an unknown specimen to a member of the complex. However, rapid fragment-based assays should also be developed that can be deployed more quickly and with more limited molecular laboratory resources. The establishment of one invasive member of a cryptic species complex should not immediately reduce vigilance on the part of prevention and quarantine officials since additional members of the complex may yet invade.

## Conclusion

*Scirtothrips dorsalis* is a species complex comprised of a minimum of 9 cryptic and 2 morphologically distinguishable species, most of which are regionally endemic. Several cryptic species within the complex appear to be invasive plant pests at different stages of anthropogenic range expansion. South Asia 1 has the largest global distribution, having expanded from its native range (which includes India) westward to Texas, USA and eastward to Japan. East Asia 1 is native to Japan, and South Korea but is newly invasive in New York, USA. South Asia 2 may also be at an earlier stage of invasion with possible invasive populations in Taiwan and Japan though this requires further corroboration. The existence of multiple morphologically indistinguishable invasive species raises practical concerns regarding invasive species detection, monitoring and prevention. Furthermore, when those species hail from different eco-climatic regions, it challenges accurate forecasting of invasion and range expansion potential based on temperature. The species delimiting framework utilized was based on 1) extensive DNA barcode library development, 2) histogram analysis of pair-wise distances and 3) the multi-species coalescent to resolve contentious species boundaries. Our results should prove useful to those identifying members of the *S*. *dorsalis* complex and our framework may also provide a practical model for delimiting other cryptic species complexes.

## Supporting Information

S1 Fig *Scirtothrips dorsalis* species complex phylogenies. a) COI gene tree, b) 28S-D2 gene tree, c) combined two-locus phylogeny with COI third codon position sites removed. The solid arrow denotes the unsubstantiated South Asia 3 cryptic species. Nodal support is the posterior probability and asterisks denote splits in >99.9% of trees.
(PDF)

S2 Fig *Scirtothrips dorsalis* species complex gene trees for a) ESRP1_2, b) DCR1, c) POLD1, d) HZF, e) htpG, f) TIF31, and g) CAD nuclear loci. Nodal support is the posterior probability and asterisks denote splits in >99.9% of trees. All trees are rooted between South Asia I-II and S. *aff*. *dorsalis*. The scale bar at the base of each tree corresponds to 0.005 substitutions/site for coding only loci (e-g) and to 0.01 substitutions per site for loci with introns (a-d).
(PDF)

S1 File File includes Tables A, B, C, and D. Table A: *Scirtothrips* barcoded for this study. Table B: *Scirtothrips* mined data (DNA sequence, host, and location). Table C: Barcode primers. Table D: Nuclear loci, KEGG orthology (KO) and annealing temperature (T_A_) used for *Scirtothrips dorsalis* complex species delimitation and phylogeny inference.
(DOCX)
