# Supplementary figures and images for: The Scirtothrips dorsalis Species Complex: Endemism and Invasion in a Global Pest
Source: PLoS One. 2015 Apr 20;10(4):e0123747. doi: 10.1371/journal.pone.0123747 (PMC4404325; doi:10.1371/journal.pone.0123747)

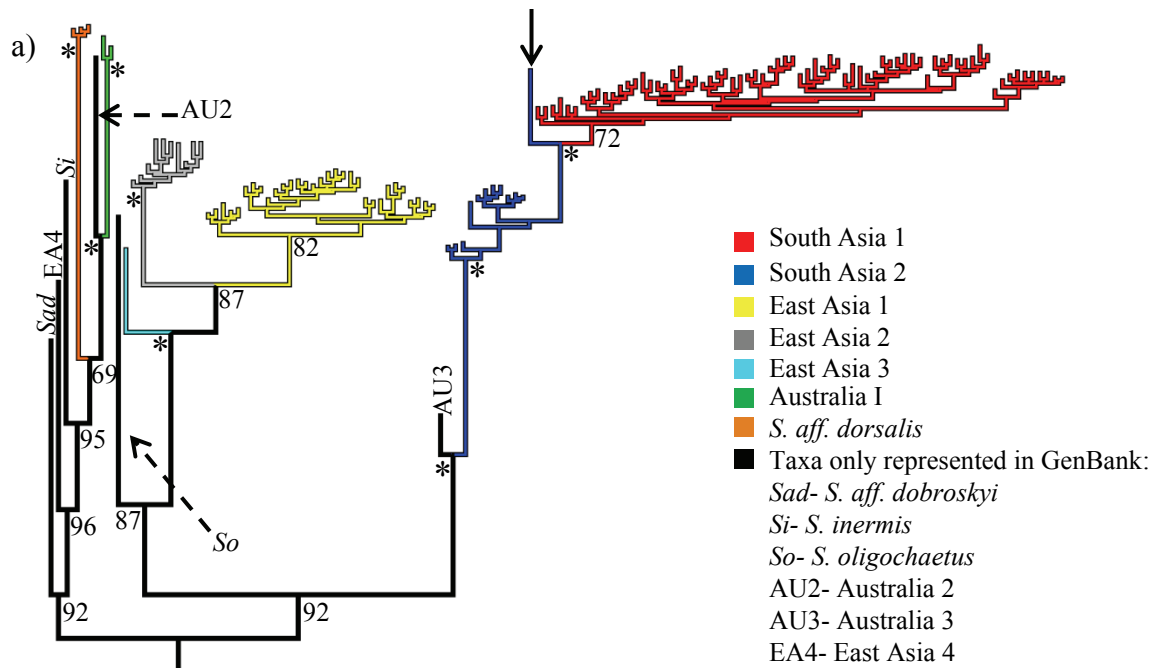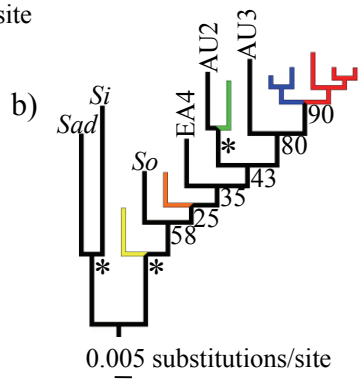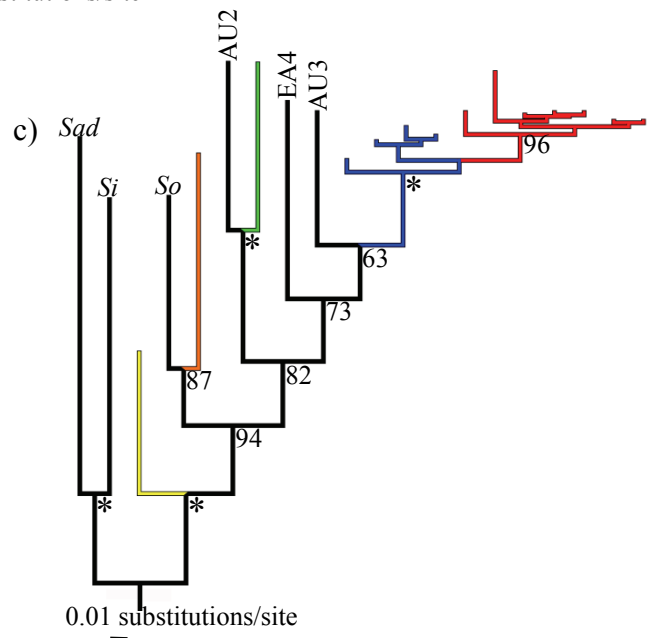

Supplement: S1 Fig — a) COI gene tree, b) 28S-D2 gene tree, c) combined two-locus phylogeny with COI third codon position sites removed. The solid arrow denotes the unsubstantiated South Asia 3 cryptic species. Nodal support is the posterior probability and asterisks denote splits in >99.9% of trees. (PDF) [file pone.0123747.s001.pdf]

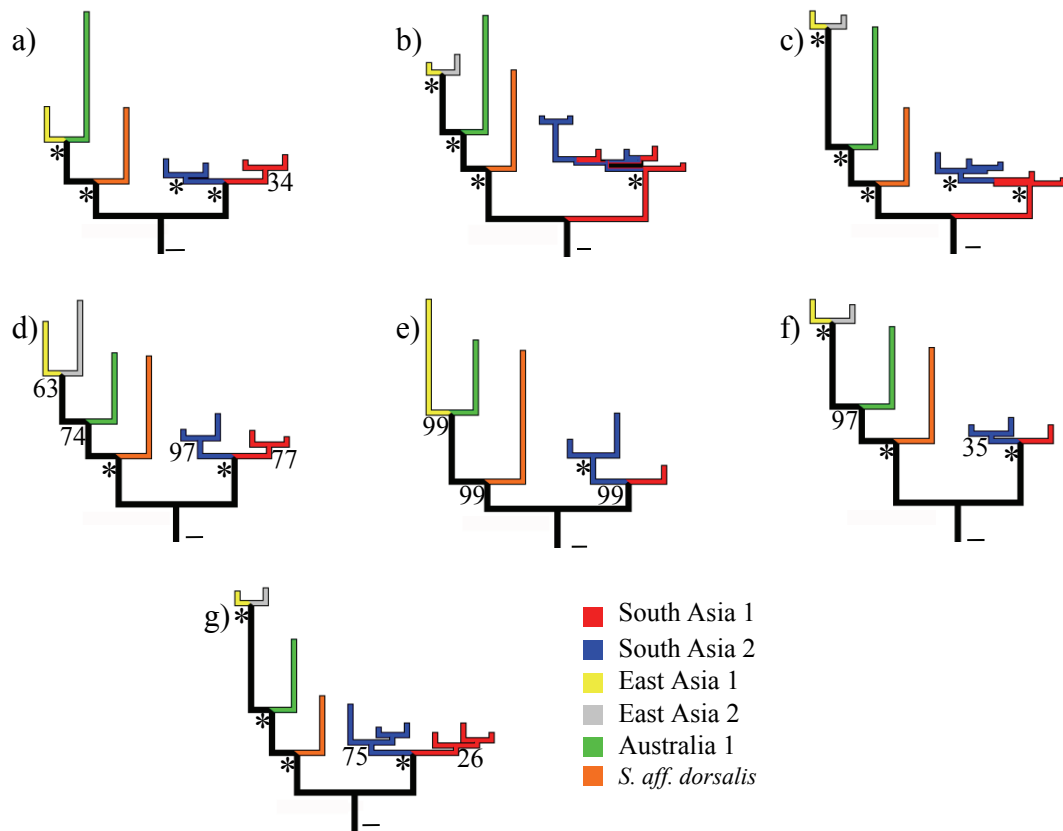

Supplement: S2 Fig — Nodal support is the posterior probability and asterisks denote splits in >99.9% of trees. All trees are rooted between South Asia I-II and S. aff. dorsalis. The scale bar at the base of each tree corresponds to 0.005 substitutions/site for coding only loci (e-g) and to 0.01 substitutions per site for loci with introns (a-d). (PDF) [file pone.0123747.s002.pdf]
