# Supplementary material for: The Scirtothrips dorsalis Species Complex: Endemism and Invasion in a Global Pest
Source: PLoS One. 2015 Apr 20;10(4):e0123747. doi: 10.1371/journal.pone.0123747 (PMC4404325; doi:10.1371/journal.pone.0123747)
Supplement: S1 File — Table A: Scirtothrips barcoded for this study. Table B: Scirtothrips mined data (DNA sequence, host, and location). Table C: Barcode primers. Table D: Nuclear loci, KEGG orthology (KO) and annealing temperature (TA) used for Scirtothrips dorsalis complex species delimitation and phylogeny inference. (DOCX) [file pone.0123747.s003.docx]

**Table A. *Scirtothirps* samples barcoded for this study.**

| Code | Location | Host Plant | Collector | Date Collected | Sample Size (*n*)- Barcoded | GenBank Accessions -KM355… | *n*- Sequenced at Nuclear Genes | GenBank Accessions - KM363… | *n*- Vouchers | *n*- Voucher Barcoded | Species |
| --- | --- | --- | --- | --- | --- | --- | --- | --- | --- | --- | --- |
| 1 | Maharashtra State, India (Geographic coordinates unavailable) |  | Jeetendra Jadhav | before 6-Apr-09 | 10 | 494-503 |  |  | 0 | 0 | South Asia 1 |
| 2 | Karamadai, Coimbatore, Tamil Nadu, India, 11 N, 77 E | *Capsicum annuum* | D. Kubendran | 29-Aug-08 | 20 | 505-522 |  |  | 5 | 0 | South Asia 1 |
| 3 | Lakhanpura, Maheshwar, Madhya Pradesh, India, 22 N, 76 E | *Capsicum annuum* var. Daftari 27 | Ajay Anandram Gahlot | 18-Nov-08 | 27 | 472-493 |  |  | 0 | 0 | South Asia 1 |
| 4 | Miyagam, Karjan, Gujarat, India, 22 N, 73 E | *Capsicum annuum* var. Sitara |  | 12-Apr-08 | 13 | 464-471 | 2 | 775,782,788,795, 801,808,814,821, 828,835,842,849, 856,863,870 | 2 | 1 | South Asia 1 |
| 5 | Bsor Research Station, NW Negev Desert, Israel, 31.272161 N, 34.387358 E | *Lisianthus sp.* | Itai Opatovsky | before 21-May-09 | 13 | 523-525 |  |  | 3 | 2 | South Asia 1 |
| 6 | Bsor Research Station, NW Negev Desert, Israel, 31.271108 N, 34.385786 E | *Lisianthus sp.* | Phyllis G. Weintraub | 27-Apr-08 | 13 | 453-454 | 1 | 774,787,800,813, 827,841,855,869 | 0 | 0 | South Asia 1 |
| 7 | Tropical Vegetable Research Center, Kasetsart University, Nakhon Pathom, Thailand, 14.0 N, 100.0 E | *Capsicum annum* var. Bangchang 365 | Jutarat Lidjan | 19-Sep-12 | 10 | KM355537-539; KM593276 | 1 | 773,786,799,812, 826,840,854,868 | 4 | 2 | South Asia 1 |
| 8 | Bangkok, Thailand (Intercepted in Transit), Origin Unknown^1^ | *Euphorbia sp.* "Millii", *Adenium obesum* | Thailand Dept. of Agriculture | 30-Jun-05 | 29 | 547-548 |  |  | 0 | 0 | South Asia 1 |
| 9 | Sashiki, Nanjyo City, Okinawa Pref., Japan, 26.2 N, 127.8 E | *Mangifera indica* | Masashi Nohara | 2-Sep-13 | 4 | 428 | 1 | 772,785,798,811, 825,839,853,867 | 3 | 3 | South Asia 1 |
| 10 | Tainan city, Taiwan, 23.16192 N, 120.447364 E | *Mangifera indica* | F. C. Lin | 15-Aug-13 | 14 | KM355420-423; KM593274,275 | 1 | KM363779,792, 805,818,832,846, 860,874;KM527189, 195,197,199,201, 203,205,207 | 4 | 2 | South Asia 1&2 |
| 11 | Botany Garden, Singapore, 1.3 N, 103.8 E | *Rosa sp.* | He Liansheng | 16-Oct-07 | 32 | 435 | 1 | KM527190,196, 198,200,202,204, 206,208 | 2 | 1 | South Asia 1 |
| 12 | Ft. Canning Park, Singapore, 1.3 N, 103.8 E | *Chrysanthmum sp.* | He Liansheng | 24-Oct-07 | 10 | 437-438 |  |  | 0 | 0 | South Asia 1&2 |
| 13 | 6 Perahu Road, Singapore, 1.4 N, 103.7 E | *Capsicum annuum* | He Liansheng | 22 & 23-Oct-07 | 23 | 436 |  |  | 0 | 0 | South Asia 1 |
| 14 | PMU, AVA, Singapore, 1 N, 104 E | *Rosa sp.* | He Liansheng | 23-Oct-07 | 1 | 440 |  |  | 0 | 0 | South Asia 1 |
| 15 | MREC Greenhouses, Apopka, Florida, USA, 28.64 N, 81.55 W | *Rosa x* 'Radrazz' | Lance Osborne | 7-Aug-07 | 22 | 526-527 |  |  | 0 | 0 | South Asia 1 |
| 16 |  | *Raphiolepis indica* |  |  | 34 | 528,536 |  |  | 0 | 0 |  |
| 17 |  | *Viburnum suspensum* |  |  | 5 | 418 |  |  | 0 | 0 |  |
| 18 |  | *Gossypium sp.* |  |  | 11 | 439 |  |  | 0 | 0 |  |
| 19 |  | *Capsicum annum* |  |  | 11 | 461 |  |  | 0 | 0 |  |

Vouchers to be deposited at the Florida State Collection of Arthropods, Gainesville

**Table A continued. *Scirtothirps* samples barcoded for the current study.**

| Code | Location | Host Plant | Collector | Date Collected | Sample Size (*n*)- Barcoded | GenBank Accessions -KM355… | *n*- Sequenced at Nuclear Genes | GenBank Accessions - KM363… | *n*- Vouchers | *n*- Voucher Barcoded | Species |
| --- | --- | --- | --- | --- | --- | --- | --- | --- | --- | --- | --- |
| 20 | MREC Greenhouses, Apopka, Florida, USA, 28.64 N, 81.55 W | *Capsicum annum* var. Chilly | Lance Osborne | 7-Aug-07 | 16 | 504 |  |  | 0 | 0 | South Asia 1 |
| 21 |  | *Ocimum basilicum* |  |  | 23 | 452 |  |  | 0 | 0 |  |
| 22 | MREC Insectary, Apopka, Florida, USA, 28.64 N, 81.55 W | *Ricinus communis* | Lance Osborne | 5-Nov-13 | 2 | 429-430 | 2 | 776,781,789,794, 802,807,815,820, 829,834,843,848, 857,862,871,876 | 3 | 1 | South Asia 1 |
| 23 | CREC, Lake Alfred, Florida, USA, 28.10 N, 81.71 W | *Citrus sp.* | Lance Osborne | before 3-Sep-08 | 11 | 455 |  |  | 0 | 0 | South Asia 1 |
| 24 | Plant City, Florida, USA, 28.04 N, 82.19 W | *Fragaria x ananassa* | Joe Funderburk | 27-Mar-08 | 9 | 451 |  |  | 0 | 0 | South Asia 1 |
| 25 | Zellwood, Florida, USA, 28.732519 N, 81.628224 W | *Vaccinium sp.* | Lance Osborne | 13-Aug-08 | 44 | 456 |  |  | 0 | 0 | South Asia 1 |
| 26 | Fort Pierce, FL, USA, 27.44 N, 80.40 W | *Schefflera arbicola* | John Prokop | 24-Oct-08 | 11 | 460 |  |  | 0 | 0 | South Asia 1 |
| 27 | USDA Greenhouses, Fort Pierce, FL, USA, 27.427 N, 80.410 W | *Citrus sp.* | Joe Albano | before 8-Dec-08 | 5 | 462-463 |  |  | 0 | 0 | South Asia 1 |
| 28 | Unicorn Island, Ben Tre Prov., Vietanam, 10.34161 N, 106.34742 E | undetermined | Mark Hoddle | 22-Aug-12 | 5 | 541-545 | 2 | 770,771,783,784, 796,797,809,810, 823,824,837,838, 851,852,865,866 | 7 | 5 | South Asia 2 |
| 29 | An Thoi Village, Ben Tre Prov., Vietnam, 10.06561 N, 106.36515 E | *Mangifera sp.* | Mark Hoddle | 21-Aug-12 | 1 | 540 |  |  |  |  | South Asia 1 |
| 30 | Iwata, Shizuoka Pref., Japan, 34.7 N, 137.8 E | *Camellia sinensis* | Shimichi Masui | 27-Aug-10 | 15 | 529-535 | 1 | 778,791,804,817, 831,845,859,873 | 1 | 0 | East Asia 1 |
| 31 | Yuki, Ibaraki Pref., Japan, 36.2 N, 139.8 E | *Camellia sinensis* | Tomoe Akiyama | 4-Sep-08 | 14 | 457-459 |  |  | 0 | 0 | East Asia 1 |
| 32 | Seogwipo city, Jeju, S Korea, 33 N, 127 E | *Citrus sp.* | Hyun Jae-Wook | 18-Mar-13 | 7 | KM355431-434; KM593278-280 |  |  | 7 | 6 | East Asia 1&2 |
| 33 | Chinese Tea Research Institute, Meijia Wu, Hangzhou, China, 30.2 N, 120.1 E | *Camellia sinensis* | Shu-Sheng Liu | 2-Oct-07 | 20 | 441-450 | 1 | 822,836,850,864 | 0 | 0 | East Asia 2&3 |
| 34 | Emerald, Central Queensland, Australia, 23.5 S, 148.1 E | *Macadamia sp.* | M. Matthews | 22-Mar-11 | 4 | 426-427 | 1 | 777,790,803,816, 830,844,858,872 | 4 | 2 | Australia 1 |
| 35 | Nelspruit, Mpumalanga, S Africa, 25.462472 S, 30.984944 E | *Ricinus communis* | Peter Stephen | Aug-13 | 4 | KM355424-425; KM593277 | 1 | 780,793,806,819,  833,847,861,875 | 0 | 2 | *S. aff. dorsalis* |
| 36 | Tuol Sleng Prison Yard, Phnom Penh, Cambodia, 11.54993 N, 104.91750 E | *Mangifera sp.* | Mark Hoddle | 31-Aug-12 | 1 | 546 |  |  | 1 | 0 | South Asia 2 |

Vouchers to be deposited at the Florida State Collection of Arthropods, Gainesville

^1^DNA courtesy of Roxanne Farris, see [12]

**Table A continued. *Scirtothirps* samples barcoded for the current study.**

| Code | Location | Host Plant | Collector | Date Collected | Sample Size (*n*)- Barcoded | GenBank Accessions -KM355… | *n*- Sequenced at Nuclear Genes | GenBank Accessions - KM363… | *n*- Vouchers | *n*- Voucher Barcoded | Species |
| --- | --- | --- | --- | --- | --- | --- | --- | --- | --- | --- | --- |
| 37 | San Antonio, Texas, USA, 29.537 N, 98.441 W | *Rosa sp.* | Ed Bradley | 29-Jul-13 | 1 | 419 |  |  | 0 | 0 | South Asia 1 |
| 38 | Riverhead, New York, USA, 40.9 N, 72.7 W | *Hydrangea sp.* | Dan Gilrein | 12-Jun-14 | 2 | KM527191,193 | 2 | KM527192,194 | 4 | 1 | East Asia 1 |

Vouchers to be deposited at the Florida State Collection of Arthropods, Gainesville

**Table B. *Scirtothrips* mined data (DNA sequence, host, and location).**

| Code | Location | Reference | Host | GenBank Accessions | Species | Voucher |
| --- | --- | --- | --- | --- | --- | --- |
| 39 | Kunurra, Western Australia | [7] | *Khaya sp.* | EU100987, EU100958 | Australia 2 | UCR |
| 40 | Kunurra, Western Australia | [7] | *Sesbania sp.* | EU100986, EU100957 | Australia 3 | UCR |
| 30 | Iwata, Shizuoka Pref., Japan, 34.7 N, 137.8 E | Dickey et al. unpublished | *Camellia sinensis* | KM349826 | East Asia 1 |  |
| 41 | Chiran, Kagoshima Pref., Japan | [10] | *Camellia sinensis* | AB818051 | East Asia 1 |  |
| 42 | Niyodogawa, Kochi Pref., Japan | [10] | *Camellia sinensis* | AB818044 | East Asia 1 |  |
| 43 | Minamishimabara, Nagasaki Pref., Japan | [10] | *Citrus unshiu* | AB818034 | East Asia 1 |  |
| 44 | Kawaminami, Miyazaki Pref., Japan | [10] | *Camellia sinensis* | AB818034 | East Asia 1 |  |
| 45 | Nankoku, Kochi Pref., Japan | [10] | *Diospyros kaki* | AB818052 | East Asia 1 |  |
| 46 | Unzen, Nagasaki Pref., Japan | [10] | *Fragaria sp.* | AB818049 | East Asia 1 |  |
| 47 | Morioka, Iwate Pref., Japan | [10] | *Malus pumila* | AB818050 | East Asia 1 |  |
| 48 | Amami, Kagoshima Pref., Japan | [10] | *Mangifera indica* | AB818038, AB818039, AB818042, AB818045 | East Asia 1 |  |
| 49 | Tokunoshima, Kagoshima Pref., Japan | [10] | *Mangifera indica* | AB818038 | East Asia 1 |  |
| 50 | Satsuma, Kagoshima Pref., Japan | [10] | *Mangifera indica* | AB818040 | East Asia 1 |  |
| 51 | Tarumizu, Kagoshima Pref., Japan | [10] | *Mangifera indica* | AB818037, AB818041 | East Asia 1 |  |
| 52 | Yoron, Kagoshima Pref., Japan | [10] | *Mangifera indica* | AB818046, AB818045 | East Asia 1 |  |
| 53 | Nago, Okinawa Pref., Japan | [10] | *Camellia sinensis* | AB818042, AB818045 | East Asia 1 |  |
| 54 | Kahoku, Ishikawa Pref., Japan | [10] | *Vitis sp.* | AB818035, AB818047 | East Asia 1 |  |
| 55 | Kofu, Yamanashi Pref., Japan | [10] | *Vitis sp.* | AB818033, AB818053 | East Asia 1 |  |
| 56 | Okinawa, Okinawa Pref., Japan | [10] | *Vitis sp.* | AB818045, AB818048 | East Asia 1&2 |  |
| 57 | Habikino, Osaka Pref., Japan | [10] | *Vitis sp.* | AB818033, AB818032 | East Asia 1 |  |
| 58 | Higashihiroshima, Hiroshima Pref., Japan | [10] | *Hydrangea macrophylla* | AB818032 | East Asia 1 |  |
| 59 | Okinawa Pref., Japan | [7] | *Erythrina sp.* | EU101007, EU100978 | East Asia 4 | UCR |
| 60 | Chiang-Mai, Thailand | [7] | *Citrus sp.* | EU100991 | Possible Numt | UCR |
| 61 | Raiatea, French Polynesia | [7] | *Persea americana* | EU100997, EU100968 | *S. aff. dobroskyi* | UCR |
| 62 | Limpopo Prov., S. Africa | [7] | *Ricinus communis* | EU100985 | *S. aff. dorsalis* | UCR |
| 63 | Kangaroo Island, Australia | [7] | *Prunus persica* | EU101002, EU100973 | *S. inermis* | UCR |
| 64 | Bangalore, India | [7] | *Mangifera indica* | EU100992, EU100963 | *S. oligochaetus* | UCR |
|  | - | [5] | *Arachis, Gossypium, Pisum, Prosopis, Punica, Solanum* |  | *S. oligochaetus* |  |
| 15 | MREC Greenhouses, Apopka, Florida, USA, 28.64 N, 81.55 W | Dickey et al. unpublished | *Rosa x 'Radrazz'* | KM349827 | South Asia 1 |  |
| 65 | Bangalore, India | [7] | *Mangifera indica* | EU100984, EU100955 | South Asia 1 | UCR |
| 66 | Chiang-Mai, Thailand | [7] | *Mangifera indica* | EU100990, EU100961 | South Asia 1 | UCR |
| 67 | Taichung, Taiwan | [7] | *Vitis sp.* | EU100988, EU100959 | South Asia 1 | UCR |
| 68 | Hainan Prov., China | Huang et al. unpublished | *Bombax malabaricum* | GU570437 | South Asia 1 |  |
| 69 | Gulberga, India | [11] | *Capsicum annuum* | HQ377270 | South Asia 1 |  |
| 70 | Minamikyusyu, Kagoshima Pref., Japan | [10] | *Mangifera indica* | AB818022 | South Asia 1 |  |
| 71 | Itoman, Okinawa Pref., Japan | [10] | *Capsicum annuum* | AB818020 | South Asia 1 |  |
| 72 | Itoman, Okinawa Pref., Japan | [10] | *Leucaena leucocephala* | AM818025 | South Asia 1 |  |
| 73 | Miyako, Okinawa Pref., Japan | [10] | *Mangifera indica* | AB818020, AB818024 | South Asia 1 |  |
| 74 | Okinawa, Okinawa Pref., Japan | [10] | *Camellia sinensis* | AB818024 | South Asia 1 |  |
| 75 | Manila, Philippines | [10] | *Capsicum sp.* | AB818022 | South Asia 1 |  |
| 76 | Kanjanaburi, Thailand | [10] | *Capsicum sp.* | AB818027 | South Asia 1 |  |
| 77 | Chiang-Mai, Thailand | [7] | Unidentified herb | EU100989, EU100960 | South Asia 2 | UCR |
| 68 | Hainan Prov., China | Huang et al. unpublished | *Camellia sinensis, Litchi chinensis, Arachis hypogaea, Persea americana, Acacia confusa, Annona squamosa, Capsicum annuum, Dimocarpus longan, Coffea arabica* | GU570431-GU570436, GU570438-GU570440 | South Asia 2 |  |
| 78 | Ishigaki, Okinawa Pref., Japan | [10] | *Mangifera indica* | AB818023 | South Asia 2 |  |

UCR-University of California Riverside Insect Collection

**Table C. Barcode primers**

| Left Primer^1^ | Sequence 5'-3' | Right Primer^1^ | Sequence 5'-3' | Source (Left, Right) | Annealing Temperature | Note |
| --- | --- | --- | --- | --- | --- | --- |
| LR-N13002 | TTACCTTAGGGATA ACAGCGT | C1-N2776 | GGTAATCAGAGTAT CGWCGNGG | (This Study*, [27]) | 54°C | Nested PCR: large, intermediate, and small products respectively. Nesting amplified all cryptic species tried (East Asia 3 not tried). |
| LR-N12866 | TACATGATTTGAGTT AAGACCGG | C1-N2189 | CAGGCAAGATTAAA ATATAAACTTCTG | (This Study, [87]) | 53°C |  |
| C1-J1514a | TTTCAACAAATCAT AAAGACATTGG | C1-N2173a | TAAACTTCAGGGTC ACCAAARAATCA | This Study† | 54°C |  |
| C1-J1514b | GGTCAACAAATCAT AAAGATATTGG | C1-N2189 | CAGGCAAGATTAAA ATATAAACTTCTG | ([26], [87]) | 56°C | The annealing temperature was 45° for the first 6 cycles. It amplified South Asia 1&2, and East Asia 1, 2&3 cryptic species. |
| C1-J1514b | GGTCAACAAATCAT AAAGATATTGG | C1-N2173c | TAAACTTCAGGGTG ACCAAAAAATCA | [26] | 54°C | Numts were amplified in East Asia 2 cryptic species with annealing temperatures between 40° and 48°. South Asia 1&2 and rarely East Asia were amplified. |
| LR-N12871 | CCAATAAAACCTTA CATGATTTGAGTTA AG | C1-N2638 | GTAAATAAAGGATA TCAATGAACTACAC CTGC | This Study | 54°C | Designed to amplify a 1300 base-pair barcode for the common invasive haplotype; it amplified several haplotypes from South Asia 1&2 (*S. aff. dorsalis* at 40°C). |
| C1-J1514a | TTTCAACAAATCAT AAAGACATTGG | C1-N2173b | TATACTTCTGGGTGA CCAAARAATCA | This Study† | 57°C | The annealing temperature was 45° for the first 6 cycles. It amplified many South Asia 1 haplotypes. |
| LR-N12866 | TACATGATTTGAGTT AAGACCGG | C1-N2353 | GCTCGTGTATCAACG TCTATNCC | (This Study, This Study*) | 57°C | The annealing temperature was 45° for the first 6 cycles. It amplified most East Asia 2&3 haplotypes and some South Asia 1 haplotypes. |
| LR-N12843 | *TGTAAAACGACGGCC* GTAAGCCAGGTTGGT TTCTATC | C1-N2173m | *CAGGAAACAGCTATG AC*TCTGGGTGACCA AAAAATCA | (This Study, This Study†) | 50°C | The primer ratios within the cocktail were Left:Right=1:1 and the M-13 tail is shown in italics, see [28]. The annealing temperature was 54° for the last 36 cycles. It amplified haplotypes from South Asia 1&2, East Asia 1, and Australia 1 but Australia 1 was amplified better without temperature stepping. |
|  |  | C1-N2173n | *CAGGAAACAGCTATG AC*TCTGGGTGACCA AARAATCA |  |  |  |
| LR-N13000 | TTACCTTAGGGATA ACAGMGTTA | C1-N2737 | AAATGTTGTGGAAA AAAYGT | (This Study*, This Study) | 51°C | The annealing temperature was 45° for the first 6 cycles. It amplified the longest barcode for the common invasive haplotype (South Asia 1). |

^1^Primer naming convention follows [27]; Gene (LR-Large Subunit Ribosomal RNA, C1-Cytochrome Oxidase I)-Majority(J) or Minority(N) strand and location in the ancestral arthropod genome. Lowercase letters denote unique primers at the same location.

*Modified from [27], †Modified from [28]

**Table S3 Continued. Barcode primers**

| Left Primer^1^ | Sequence 5'-3' | Right Primer^1^ | Sequence 5'-3' | Source (Left, Right) | Annealing Temperature | Note |
| --- | --- | --- | --- | --- | --- | --- |
| C1-J1505a | *TGTAAAACGACGGCC* GACTTTTTTCTTCAA ATCATAA | C1-N2173m | *CAGGAAACAGCTATG AC*TCTGGGTGACCA AAAAATCA | (This Study, This Study†) | 54°C | The annealing temperature was 50° for the first 6 cycles.The primer ratios within the cocktail were Left:Right=1:1. The M-13 tail is shown in italics, see [28]. It confirmed and/or extended a few South Asia 1&2 haplotypes and *S. aff. dorsalis*. |
| C1-J1505b | *TGTAAAACGACGGCC* GACTTTTTTCTTCAA AYCAYAA | C1-N2173n | *CAGGAAACAGCTATG AC*TCTGGGTGACCA AARAATCA |  |  |  |
| C1-J1514f | *TGTAAAACGACG*TTT TCTTCAAATCATAA AGATATTGG | C1-N2173j | *CAGGAAACAGCTATG AC*TTCAGGATGTCC AAAAAATCA | This Study† | 54°C | The annealing temperature was 50° for the first 6 cycles. The primer ratios within the cocktail were a) Left:Right=1:1, and b) Containing inosine:Lacking inosine=3:2. The M-13 tail is shown in italics, see [28]. It confirmed and/or extended a few South Asia 1&2 haplotypes. |
| C1-J1514g | *TGTAAAACGACG*TTT TCTTCAAATCAYAA AGAYATTGG | C1-N2173k | *CAGGAAACAGCTATG AC*TTCAGGATGTCC AAARAATCA |  |  |  |
| C1-J1514h | *TGTAAAACGACG*TTT TCTTCAAATCAIAA AGAIATIGG | C1-N2173l | *CAGGAAACAGCTATG AC*TTCAGGATGICCI AAIAAICA |  |  |  |
| C1-J1514c | *TGTAAAACGACGGCC AG*TTTTCTTCAAAT CATAAAGATATTGG | C1-N2173f | *CAGGAAACAGCTATG AC*TAAACTTCTGGG TGICCAAAIAAICA | This Study† | 54°C | The annealing temperature was 50° for the first 6 cycles. The primer ratios within the cocktail were a) Left:Right=1:1, and b) Containing inosine:Lacking inosine=3:2. The M-13 tail is shown in italics, see [28]. It confirmed and extended one East Asia 1 haplotype. |
| C1-J1514d | *TGTAAAACGACGGCC AG*TTTTCTTCAAAT CAYAAAGAYATTGG | C1-N2173g | *CAGGAAACAGCTATG AC*TAAACTTCAGGA TGTCCAAAAAATCA |  |  |  |
| C1-J1514e | *TGTAAAACGACGGCC AG*TTTTCTTCAAAT CAIAAAGAIATIGG | C1-N2173h | *CAGGAAACAGCTATG AC*TAAACTTCAGGA TGYCCAAARAATCA |  |  |  |

^1^Primer naming convention follows [27]; Gene (LR-Large Subunit Ribosomal RNA, C1-Cytochrome Oxidase I)-Majority(J) or Minority(N) strand and location in the ancestral arthropod genome. Lowercase letters denote unique primers at the same location.

*Modified from [27], †Modified from [28]

**Table S3 Continued. Barcode primers**

| Left Primer^1^ | Sequence 5'-3' | Right Primer^1^ | Sequence 5'-3' | Source (Left, Right) | Annealing Temperature | Note |
| --- | --- | --- | --- | --- | --- | --- |
| C1-J1514c | *TGTAAAACGACGGCC AG*TTTTCTTCAAAT CATAAAGATATTGG | C1-N2173d | *CAGGAAACAGCTATG AC*TAAACTTCTGGG TGACCAAAAAATCA | This Study† | 54°C | The annealing temperature was 50° for the first 6 cycles. The primer ratios within the cocktail were a) Left:Right=1:1, and b) Containing inosine:Lacking inosine=3:2. The M-13 tail is shown in italics, see [28]. It confirmed a few South Asia 1&2 haplotypes. |
| C1-J1514d | *TGTAAAACGACGGCC AG*TTTTCTTCAAAT CAYAAAGAYATTGG | C1-N2173e | *CAGGAAACAGCTATG AC*TAAACTTCTGGG TGRCCAAARAATCA |  |  |  |
| C1-J1514e | *TGTAAAACGACGGCC AG*TTTTCTTCAAAT CAIAAAGAIATIGG | C1-N2173f | *CAGGAAACAGCTATG AC*TAAACTTCTGGG TGICCAAAIAAICA |  |  |  |
| C1-J1562 | TCAGGAATACTGGGG CTGTC | C1-N2060 | CCCCTGCAAGAACAG GTAAAG | This Study | 55°C | Amplified an abbreviated barcode from invasive East Asia 1 haplotypes in New York, USA; used only to confirm longer barcodes. |
| C1-J1761 | CTGATATAGCATTTC CTCGTCTTAAT | C1-N2077 | ATTTCGGTCTGTTAA AAGTATAGTAATAG CACC | This Study | 53°C | Designed to amplify an abbreviated barcode from degraded DNA after [30] containing the common invasive haplotype (S Asia 1); it also confirmed many South Asia 1&2 haplotypes. It was only used to confirm longer barcodes as it was predicted to also amplify numts. |
| C1-J1710 | TTGGTGGATTTGGTA ATTGA | C1-N1908 | CCTGCTATGTGAAGG GAAAAA | (This Study*, This Study) | 51°C | Designed to amplify an abbreviated barcode from degraded DNA after [30] containing any known Australia 1, 2, or 3 cryptic species haplotype. It was only used to confirm longer Australia 1 barcodes. |

^1^Primer naming convention follows [27]; Gene (LR-Large Subunit Ribosomal RNA, C1-Cytochrome Oxidase I)-Majority(J) or Minority(N) strand and location in the ancestral arthropod genome. Lowercase letters denote unique primers at the same location.

*Modified from [27], †Modified from [28]

Reference (not in main text):

87. Brunner PC, Fleming C, Frey JE. A molecular identification key for economically important thrips species (Thysanoptera: Thripidae using direct sequencing and a PCR-RFLP-based approach. Agric For Entomol. 2002;4: 127-136.

**Table S4. Nuclear loci, KEGG orthology (KO), and annealing temperature (T_A_) used for *Scirtothrips dorsalis* complex species delimitation and phylogeny inference.**

| Predicted Gene (*A. pisum*) | KO | Reference | Name | Primer sequences | T_A_ |
| --- | --- | --- | --- | --- | --- |
| CAD | CAD | This Study | CAD_SD-F | CGGTACACACCAGAGCCTAT | 48 |
|  |  |  | CAD_SD-R | TGACATTCGGAATTCTGTCACT |  |
| Dicer 1 | DCR1 | This Study | DCR1_SD-F | TTCGATAATTCCTACCAATTCCTT | 48 |
|  |  |  | DCR1_SD-R | GCTGAGGATGTAGAGGTACCAAA |  |
| DNA polymerase δ subunit | POLD1 | This Study | POLD1_SD-F | TGAGTGTGCTGGAAGAAAGG | 48 |
|  |  |  | POLD1_SD-R | CACCCTACCCAAGTATGAAAAG |  |
| Protein TIF31 | TIF31 | This Study | TIF31_SD-F | TTCTCTCCCATCCAAAATACA | 44 |
|  |  |  | TIF31_SD-R | TCCAAATCTTTCTTGTCTCC |  |
| Heat shock protein 83 | htpG | This Study | htpG_SD-F | TCTGCTGCAACATTTCACG | 50 |
|  |  |  | htpG_SD-R | ACCAACGATTGGGAAGAACA |  |
| RNA-binding protein fusilli | ESRP1_2 | This Study | ESRP1_2_SD-F | GCCGTGCATTCATAGGGTAA | 44 |
|  |  |  | ESRP1_2_SD-R | TGCAGCTCAAAGAATGAAGA |  |
| Helicase with zinc finger domain | None | This Study | HZF_SD-F | AAATACTTACTTGATGGAAAAATGGA | 48 |
|  |  |  | HZF_SD-R | TCAACAAGCTGAGCAGCAC |  |
| 28S-D2 | - | [34] | 28S-D2-F | CGTGTTGCTTGATAGTGCAGC | 50 |
|  |  |  | 28S-D2-R | TTGGTCCGTGTTTCAAGACGG |  |

For the seven newly developed primers, T_A_ was lowered by 5° or to a minimum of 40° for samples from the Australia 1, *S. aff. dorsalis*, and East Asia 2 cryptic species.

Kyoto Encyclopedia of Genes and Genomes (KEGG)
